# Classification of Multiple Chinese Liquors by Means of a QCM-based E-Nose and MDS-SVM Classifier

**DOI:** 10.3390/s17020272

**Published:** 2017-01-30

**Authors:** Qiang Li, Yu Gu, Jing Jia

**Affiliations:** School of Automation and Electrical Engineering, University of Science and Technology Beijing, Beijing 100083, China; tslee@xs.ustb.edu.cn (Q.L.); s20160598@xs.ustb.edu.cn (J.J.)

**Keywords:** Chinese liquor classification, Multidimensional scaling (MDS), Support Vector Machine (SVM), QCM-based e-nose

## Abstract

Chinese liquors are internationally well-known fermentative alcoholic beverages. They have unique flavors attributable to the use of various bacteria and fungi, raw materials, and production processes. Developing a novel, rapid, and reliable method to identify multiple Chinese liquors is of positive significance. This paper presents a pattern recognition system for classifying ten brands of Chinese liquors based on multidimensional scaling (MDS) and support vector machine (SVM) algorithms in a quartz crystal microbalance (QCM)-based electronic nose (e-nose) we designed. We evaluated the comprehensive performance of the MDS-SVM classifier that predicted all ten brands of Chinese liquors individually. The prediction accuracy (98.3%) showed superior performance of the MDS-SVM classifier over the back-propagation artificial neural network (BP-ANN) classifier (93.3%) and moving average-linear discriminant analysis (MA-LDA) classifier (87.6%). The MDS-SVM classifier has reasonable reliability, good fitting and prediction (generalization) performance in classification of the Chinese liquors. Taking both application of the e-nose and validation of the MDS-SVM classifier into account, we have thus created a useful method for the classification of multiple Chinese liquors.

## 1. Introduction

Chinese liquor is one of the oldest distillates in the world, dating back thousands of years [1]. Some four million kiloliters of Chinese liquor are consumed annually, worth 500 billion Chinese Yuan (equivalent to US$80 billion) [2]. As famous drinks, Chinese liquors are usually fermented from grains for several months or years. The fresh fermented liquors are then distilled and aged for a long time to enhance the bouquet. The different brewing processes (fermentation, distillation, and aging) lead to the formation of a diverse set of components in Chinese liquor products, e.g., over 1600 compounds for Xifeng liquor, over 1800 compounds for Moutai liquor, and over 1900 compounds for Fen liquor. Chinese liquors from different plants have unique flavors attributable to the use of various bacteria and fungi, raw materials, and production processes [3]. Therefore, different brands of Chinese liquors display remarkable differences in flavor. The flavors of Chinese liquors are traditionally classified into five groups: namely strong-flavor, mixed-flavor, fen-flavor, moutai-flavor, and special-flavor. In particular, strong-flavor and mixed-flavor are the most common.

Chinese liquors labelled with false information not only harm the interests of consumers, but also damage producers’ interests [4]. The traditional and most commonly used method for the classification of Chinese liquors is by professional sommeliers, but accuracy and objectivity cannot always be ensured because sommeliers’ judgement can affected by their health condition, emotions, and the environment. Other methods for the analysis and classification of Chinese liquors are chemistry-based methods such as gas chromatography, mass spectrometry, and gas chromatography-mass spectrometry [5,6,7,8]. These methods are highly reliable because they use a complete component-by-component approach. However, their shortcomings include high cost, being time-consuming, and low capability for in situ and online measurements [9]. Overall, developinga novel, rapid and reliable method to identify multiple Chinese liquors is of positive significance.

A quartz crystal microbalance (QCM)-based electronic nose (e-nose) has been successfully utilized to detect characteristics of Chinese liquors by imitating the human senses using sensor arrays and a pattern recognition system [10]. The use of an excellent pattern recognition algorithm in the pattern recognition system is a key component for improving the performance of QCM-based e-noses. Shaffer et al. [11] summarized six qualities of the ideal pattern recognition algorithm for an e-nose: it should have high accuracy, low memory requirements, and be fast, simple to train, robust to outliers, and produce a measure of uncertainty.

Unfortunately, until now, no pattern recognition algorithm is able to fully meet all of these requirements. In an attempt to determine the optimal classifier, several researchers have performed studies comparing pattern recognition algorithms as well as specific applications. Peng et al. [12] presented discriminant models of Chinese Tongshan kaoliang liquor using principal component analysis (PCA) and discriminant factor analysis (DFA), and realized a correct prediction classification rate of 93%.

Our group has reported the design and application of a novel and simple QCM-based e-nose [13,14] for quickly and easily summarizing Chinese liquor characteristics. We identified three types of Chinese liquors on the basis of the Moving Average−Linear Discriminant Analysis (MA-LDA) algorithm, which had a prediction accuracy of 98%, and five types of Chinese liquors by means of the Principle Components Analysis−Back Propagation Neutral Network (PCA-BPNN) algorithm, which had a prediction accuracy of 93.3%. Additionally, Zhang et al. [15] used PCA incorporated with discriminant analysis (PCA-DA), a back propagation artificial neural network (BP-ANN), and learning vector quantization (LVQ) for the recognition of five Chinese liquors; the recognition accuracies of PCA-DA, BP-ANN, and LVQ were 76.8, 71.4, and 89.3%, respectively. Jing et al. [9] studied the classification of seven Chinese liquors by using BP-ANN, LDA, and a multi-linear classifier; the classification rates were 97.22, 98.75, and 100%, respectively. Lastly, Ema et al. [16] presented an odor-sensing system to identify eleven brands of liquors using six QCM resonators with different coating materials and neural network pattern recognition. However, the prediction accuracy of this system was only 88%.

In this paper, we present the used in a QCM-based e-nose we have designed of an algorithm based on Multidimensional Scaling (MDS) and Support Vector Machine (SVM). Performance was assessed through classifying ten brands of Chinese liquor samples.

## 2. Experiments and Methods

### 2.1. Chinese Liquor Samples

A total of ten experimental samples, corresponding to ten Chinese liquor brands, were obtained from the China National Research Institute of Food & Fermentation Industries (Beijing, China). The samples differed in main raw materials, fermentation starter, fermentation duration, aging duration, flavor type and geographic origin. All samples were produced in 2011, and had equivalent proofs. The Chinese liquors included in the study are listed in Table 1 (all data is from the database of China Alcoholic Drinks Association).

### 2.2. QCM-Based Sensor

**Figure 1:**
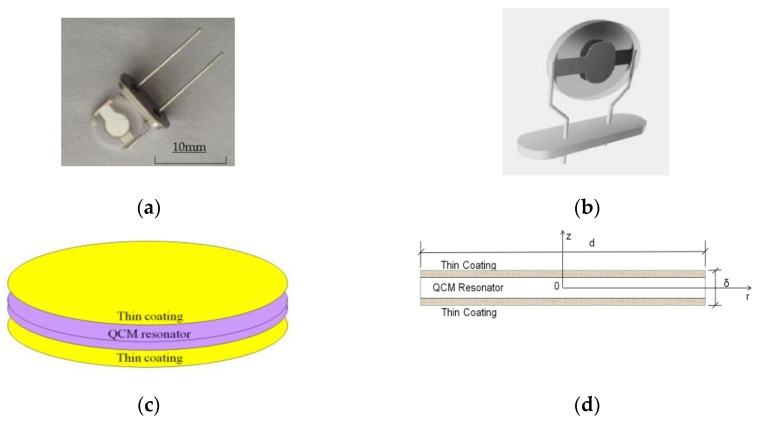
(**a**) Photo of the sensor; (**b**) structure chart of the sensor; (**c**) schematic diagram of the sensor; (**d**) diameter drawing of the sensor.

Figure 1a shows an individual QCM-based sensor; Figure 1b,c presents its structure, which had thin coatings symmetrically adhered on both sides of an AT-cut quartz piezoelectric crystal plate resonator; Figure 1d shows the diameter of the sensor. The diameter of the sensor was d = 8 mm and its thickness was δ = 0.17 mm.

The AT-cut quartz piezoelectric crystal plateresonator is an electromechanical converter that can present resonant frequency signals based on the QCM principle [19], as illustrated by Equation (1):

(1) $$\Delta f=-\frac{2{f_{0}}^{2}}{A\sqrt{\rho_{q}\mu_{q}}}\cdot \Delta m$$

where, *f*_0_ is the resonant frequency (Hz), Δ*f* is the frequency change (Hz), Δ*m* is the mass change(g), *A* is the piezoelectrically active crystal area (cm^2^), *ρ_q_* is the density of quartz (*ρ_q_* = 2.643 g/cm^3^), and *μ_q_* is the shear modulus of quartz for AT-cut crystalquartz (*μ_q_* = 2.947 × 10^11^ g·cm^−1^·s^−2^).

The thin coatings were analyte-sensitive with adsorption-desorption properties. QCMs measure the mass per unit area by measuring the change in resonator frequency of the sensor, which is disturbed by the addition or removal of mass deposited at the sensor surface. Sensor properties (selectivity, sensitivity, regenerability, cumulability) can be adjusted within wide limits by an appropriate choice of thin coating.

**Figure 2:**
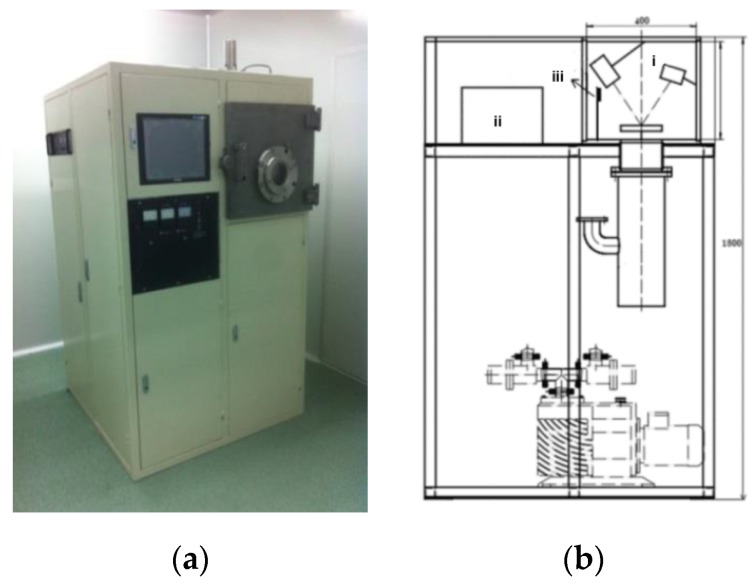
(**a**) Photo of the EBVD equipment; (**b**) schematic representation of the EBVD technology: (i) electron beam deposition system in vacuum chamber; (ii) control system; (iii) coating thickness control system.

The thin coatings were prepared by electron beam vapor dispersion (EBVD) equipment [20], as shown in Figure 2a. The EBVD equipment contained an electron beam deposition system in the vacuum chamber, a control system, and a real-time monitoring system of the thin coating’s thickness (Figure 2b).

### 2.3. QCM-Based E-Nose

**Figure 3:**
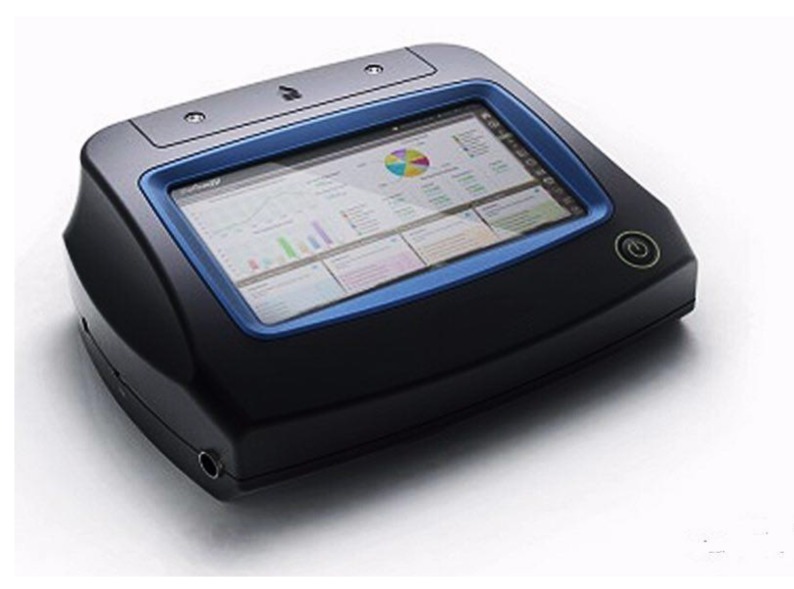
Photo of the QCM-based e-nose.

**Figure 4:**
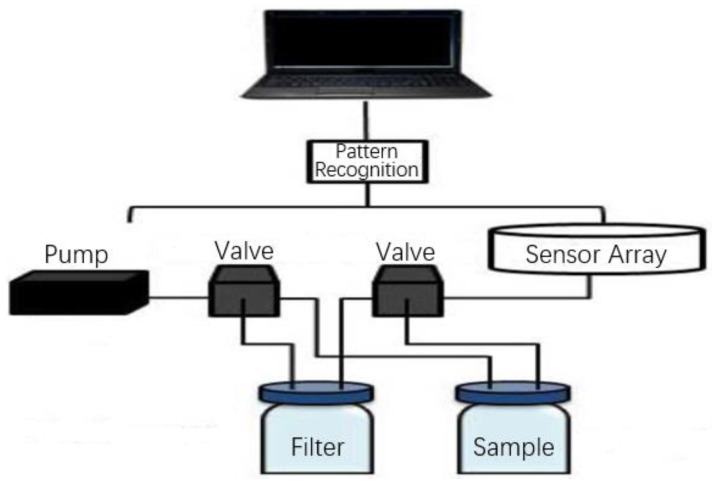
Schematic diagram of the QCM-based e-nose.

**Figure 5:**
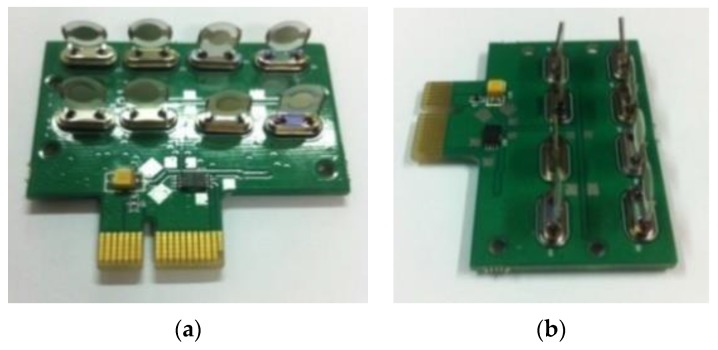
Image of the QCM sensor array. (**a**) front view; (**b**) slide view.

**Figure 6:**
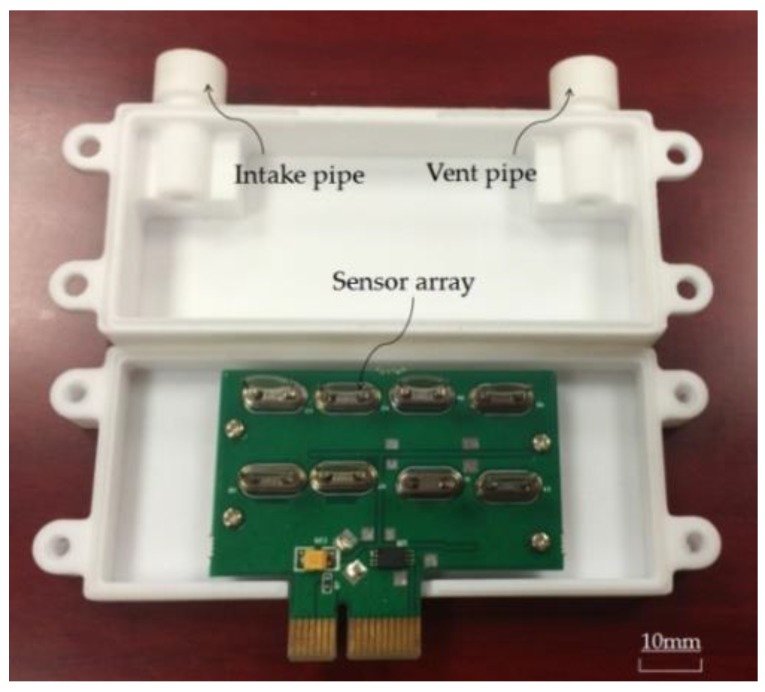
Photo of the sensor box.

In this experiment, we used a QCM-based e-nose [14] (designed as shown in Figure 3) to obtain Chinese liquors’ characteristic information, i.e., to obtain the resonator frequency signal values (RFSVs) of an eight-channel sensor array as raw data. Our e-nose was composed of three main components: (i) a gas flow system (containing a thermo-hygrostat system and an air pump), (ii) a sensor array system (containing an eight-channel gas sensor array) and (iii) an electronic circuit (containing a digital frequency counter) and pattern recognition system (Figure 4). In the gas flow system, a flow-controllable air pump was used to generate gas flow. The ambient air was used as the carrier gas to deliver the sample odor through the sensor array chamber at a flow rate of 25 mL/s. The gas-flow system was controlled by valves to switch between the filter bottle and sample bottle. The sensor array system (shown in Figure 5) consisted of eight QCM-based sensors, each of which was specially selected to detect the liquor volatiles, as listed in Table 2. The sensors were installed inside a chamber (shown in Figure 6), designed to evenly distribute the gas flow through all sensors, which was made from Teflon to prevent odor adsorption within the chamber. The electronic circuit provided output from the resonators of the eight sensors. Moreover, the data processing and visualization were conducted by the pattern recognition system.

### 2.4. Characteristic Information Acquisition by the QCM-Based E-Nose

We used ten brands of Chinese liquors (shown in Table 1) as samples in our experiments. The experiments were conducted in a clean room at a controlled temperature of 25 °C. Taking the Fen liquor (number 1 in Table 1) as an example, we firstly injected 15 mL of the Fen liquor sample into a head space bottle (volume 25 mL). Then, the e-nose was utilized for acquiring characteristic information (resonant frequency signal values) of the Fen liquor sample. The RFSVs of the sensor array were output 100 times per minute and saved. Experiments lasted for two minutes for each sample. The same process was used for the other nine liquor samples.

**Figure 7:**
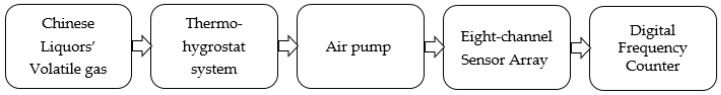
Working flow chart of the e-nose.

A working flow chart of the e-nose can be seen in Figure 7. The dryness index and temperature of Chinese liquors’ volatile gas were kept constant through the thermo-hygrostat system, while the flow velocity of the volatile gas was kept constant by the air pump.

### 2.5. Pattern Recognition System

An algorithm based on a MDS and an SVM was applied to the pattern recognition system in the QCM-based e-nose.

#### 2.5.1. Data Pre-Processing with MDS

An MDS algorithm [21], which can enhance recognition efficiency and reduce the computational burden of the QCM-based e-nose, was used for dimensionality reduction. MDS algorithms take an input matrix of dissimilarities between pairs of items and output a coordinate matrix. Min-max normalization was utilized to scale the datasets in greater numeric ranges into smaller numeric ranges to remove the limitation of data units and order of magnitudes [22].

#### 2.5.2. Classification with SVM

For the classification of pre-processed data, we have applied an SVM algorithm [23]. For nonlinear separable classification problems, the SVM applies a kernel function K (v_i_, v_j_) to transform the original space to a higher-dimensional space, and a hyper plane is constructed in the higher-dimensional space to solve problems of nonlinear separable classification in the original low-dimensional space. The four most known kernels are commonly used: linear, polynomial, radial basis function (RBF), and sigmoid.

In this work, a RBF kernel function was attempted for classification due to its good generalization. The selection of the kernel function parameter affected the precision of the SVM significantly. The optimal parameter in the kernel function was set using the particle swarm optimization (PSO) method [24].

## 3. Results and Discussion

### 3.1. Raw Data of Characteristic Information

**Figure 8:**
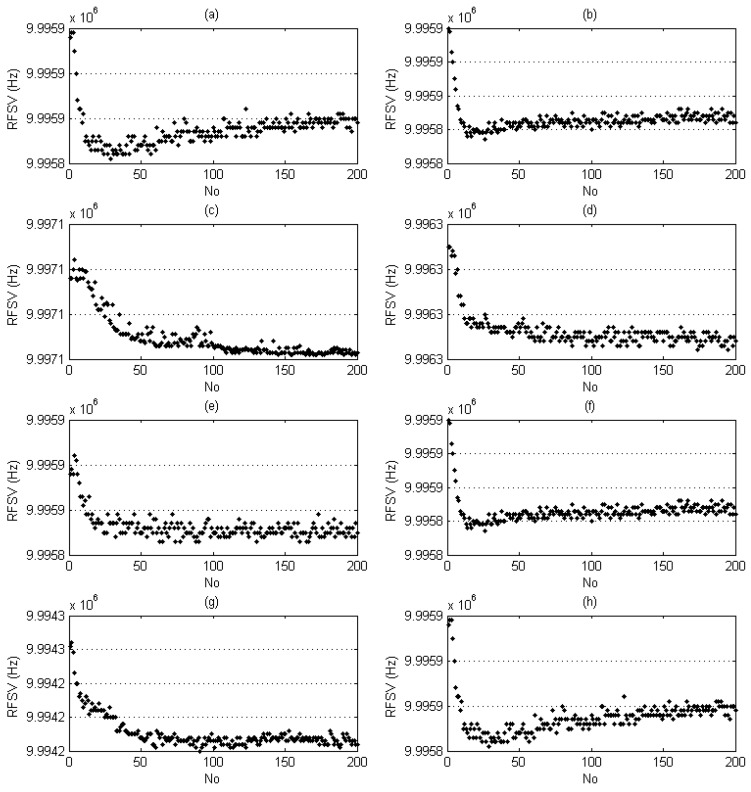
Group of raw data of the Moutai sample’s characteristic information from Sensor-1 to Sensor-8: (**a**) Sensor-1; (**b**) Sensor-2; (**c**) Sensor-3; (**d**) Sensor-4; (**e**) Sensor-5; (**f**) Sensor-6; (**g**) Sensor-7; (**h**) Sensor-8.

Taking Moutai liquor sample as example, a group of raw data, 8 × 200 RFSVs obtained by sensor-1 to sensor-8 (100 RFSVs per min for each sensor), are listed in Table 3. Their distributions are displayed in Figure 8. RFSV distributions exhibited unique magnitudes and shapes.

Each sensor in the eight-channel sensor array interacted individually with analyte because each sensor had a distinct coating. Experiments were conducted eight times, and eight groups of RFSVs were obtained. The same process was used for the other nine brands. The dataset, a total of 8 × 10 groups of raw data, was utilized to establish the pattern recognition system in the QCM-based e-nose for classifying the ten brands of Chinese liquors.

### 3.2. Liquor Classification

#### 3.2.1. Data Pre-Processing Results with MDS

Each group of raw data, 8 × 200 RFSVs, was constructed into an original sample matrix in order by:

*S*_original_ = {*S*_1,2,3,......,i_}, (10)

where, the *i*-th matrix *S_i_* is shown Equation (11):

(11) $$S_{i}=\left[\begin{array}{cccc} {f_{i}}_{{}_{1,1}} & f_{i_{1,2}} & \cdots & f_{i_{1,200}}\\ f_{i_{2,1}} & f_{i_{2,2}} & \cdots & f_{i_{2,200}}\\ \vdots & \vdots & \ddots & \vdots \\ f_{i_{8,1}} & f_{i_{8,2}} & \cdots & f_{i_{8,200}} \end{array}\right],$$

where, i={i|i∈[1,80]∩N}, and f_im,n_ represents the *n*-th RFSV acquired by the sensor *m*. For each matrix *S_i_*, MDS algorithm was used to extract a characteristic value from 200 RFSVs in each row for dimension reduction. Thus we obtained the characteristic value matrix *N_i_* for each *S_i_*:

(12) $$N_{i}=\left[\begin{array}{c} c_{i_{1}}\\ c_{i_{2}}\\ \vdots \\ c_{i_{8}} \end{array}\right],$$

where, *c_i_*_j_ (j = 1,...,8) represents the characteristic value of sensor j. Then a new sample matrix *S_new_*, 8 × 80, was obtained as Equation (13):

(13) $$S_{new}=\left\{N_{1},N_{2},N_{3},\ldots ,N_{80}\right\}$$

**Figure 9:**
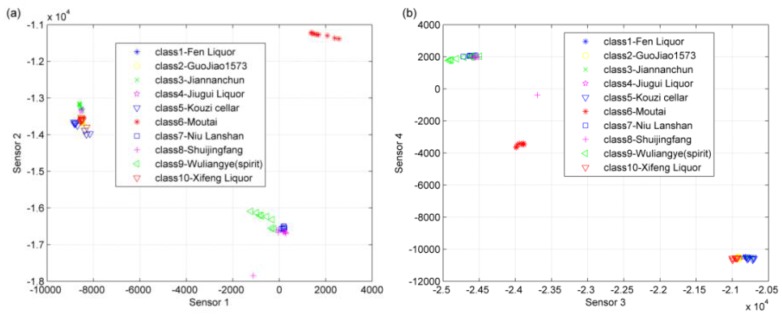

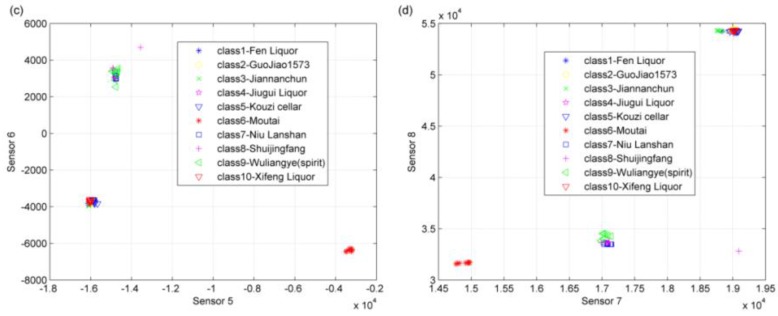
(**a**) Distribution of the 80 characteristic values for sensor 1 and sensor 2; (**b**) the distribution of the 80 characteristic values for sensor 3 and sensor 4; (**c**) distribution of the 80 characteristic values for sensor 5 and sensor 6; (**d**) distribution of the 80 characteristic values for sensor 7 and sensor 8.

The distributions of the 80 samples’ characteristic values of the eight sensors are shown in Figure 9. Many points clearly overlapped. Thus, it was difficult to classify the ten brands of Chinese liquors only on the basis of any two sensors. In the following classification process, we used characteristic information from all eight sensors, namely eight characteristic components.

Min-max normalization was employed to normalize the sample matrix *S_new_.* The values for *y*_min_ and *y*_max_ were set to zero and one. For each element in the matrix *S_new_*, *y* = (*x* − *x*_min_)/(*x*_max_ − *x*_min_) was used to calculate the new normalized value. Thus, we obtained a new sample matrix set:

(14) $$S=\text{\{}X_{1},X_{2},X_{3},\ldots \ldots ,X_{80}\text{\}},$$

where, ***S*** is an 8 × 80 matrix set. Each column represented a set of characteristic information of a Chinese liquor sample.

**Figure 10:**
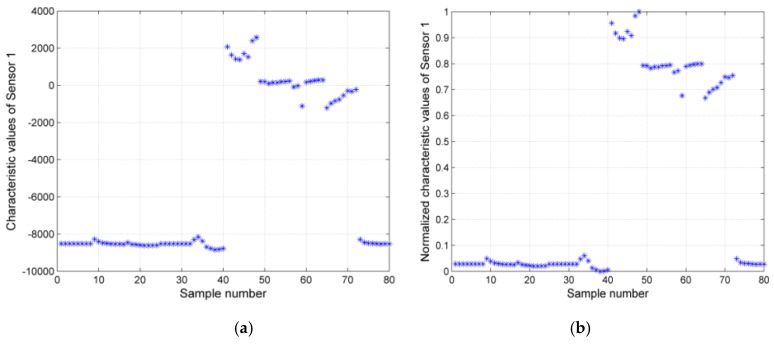

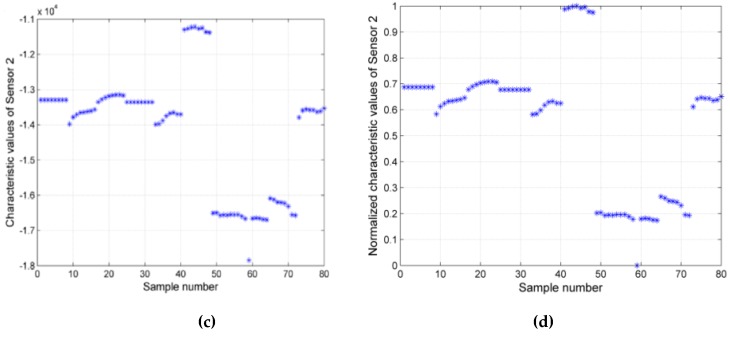
(**a**) Characteristic values the 80 samples for sensor 1; (**b**) normalized characteristic values of the 80 samples for sensor 1. (**c**) characteristic values the 80 samples for sensor 2; (**d**) normalized characteristic values the 80 samples for sensor 2.

Taking the first two sensors as an example, their characteristic values and normalized characteristic values of the 80 characteristic values are shown in Figure 10. Each asterisk in the figure represents a characteristic value, plotted by sample number on the horizontal axis, and plotted by (a) characteristic values and (b) the normalized characteristic values on the vertical axis, respectively.

#### 3.2.2. Classification Results with SVM

For matrix set ***S*** = {***X***_1_, ***X***_2_, ***X***_3_, …, ***X***_80_}, the category label was constructed in order as follows:

(15) $$\mathrm{Label}=[\overset{8}{\overset{︷}{1,\ldots ,1}},\overset{8}{\overset{︷}{2,\ldots ,2}},\overset{8}{\overset{︷}{3,\ldots ,3}},\overset{8}{\overset{︷}{4,\ldots ,4}},\ldots \ldots ,\overset{8}{\overset{︷}{9,\ldots ,9}},\overset{8}{\overset{︷}{10,\ldots ,10}}].$$

The labelled *S* was randomly split into two sections: one was used for training, and the other for testing. The SVM algorithm was used to classify the ten brands of Chinese liquor samples. We selected the RBF kernel function in the SVM algorithm, and the kernel parameter was optimized using the PSO method. To assess the performance of the established classifier, leave-one-out cross-validation and 5-fold cross-validation [25] were conducted. These cross-validations fully assessed the performance of the classification model.

**Figure 11:**
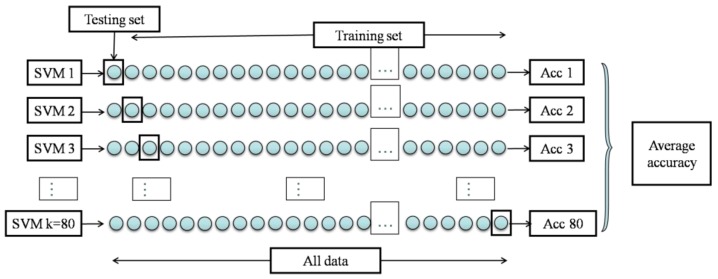
Diagram of leave-one-out cross-validation (k-fold cross-validation with k = 80).

As shown in Figure 11, for SVM classification with leave-one-out cross-validation, independent validations were conducted 80 times. In each independent validation, only one sample was selected as the testing set, and the remaining 79 samples were the training set. The average of the 80 SVM classifiers’ accuracies was regarded as the final classification accuracy of the SVM method.

**Figure 12:**
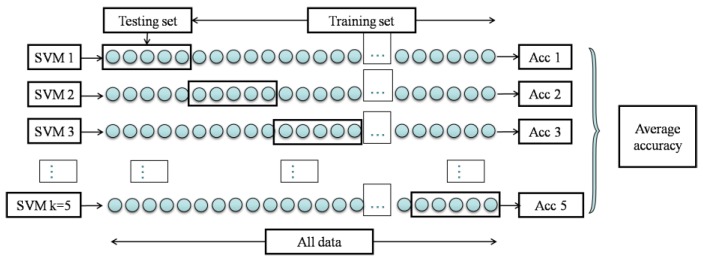
Diagram of k-fold cross-validation with k = 5.

As shown in Figure 12, for SVM classification with 5-fold cross-validation, total samples were randomly partitioned into five equally sized subsamples: a single subsample was retained for testing the classification model, and the remaining four subsamples were used as the training set. Five independent validations were conducted, with each subsample used exactly once as the testing set. The average of the five SVM classifiers’ accuracies was regarded as the final classification accuracy of the SVM method.

**Figure 13:**
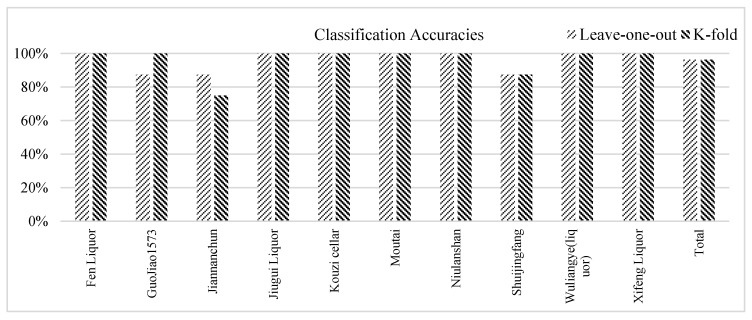
Classification accuracies of the ten brands of Chinese liquors by leave-one-out cross-validation and 5-fold cross-validation.

Figure 13 shows the classification accuracies of the ten brands of Chinese liquors by the leave-one-out cross-validation and 5-fold cross-validation, respectively. Both classification rates of our proposed MDS-SVM classifier to the QCM-based e-nose were 96.25% (77/80). Table 4 provides further details of these classification results.

### 3.3. Test Experiments

We evaluated the comprehensive performance of the MDS-SVM classifier that predicted all ten brands of Chinese liquors individually. The ten brands of Chinese liquors used as samples in our experiments, whose details are listed in Table 1, were purchased from the China National Research Institute of Food & Fermentation Industries.

The experiments were conducted in a clean room at a controlled temperature of 25 °C. Taking the Fen liquor as an example, we firstly injected 15 mL of the Fen liquor sample into ahead space bottle (volume 25 mL). Then, the e-nose was utilized for acquiring the RFSVs of the Fen liquor sample. The RFSVs were processed by the proposed MDS-SVM classifier. We repeated the experiment with the same brand of Chinese liquor sample 30 times. The same processes were conducted for the other nine brands of Chinese liquor samples.

Table 5 presents the overall accuracy for each brand of the Chinese liquors. In predicting all ten samples, the highest performing predictions are obtained for the Fen Liquor, Jiugui Liquor, Kouzi, Moutai, Niulanshan, Wuliangye and Xifeng Liquor, with overall accuracies of 100%, while, the prediction accuracies of the MDS-SVM classifier on classification of GuoJiao1573, Jiannanchun and Shuijingfang are 93.4, 93.4 and 96.7%, respectively. The average predicted accuracy for all ten brands is 98.3%. The experiments indicate that the MDS-SVM classifier has reasonable reliability, good fitting and prediction (generalization) performance in the classification of the Chinese liquors.

### 3.4. Comparison of Classification Ability

Table 6 shows the classification ability of the MDS-SVM method we proposed in terms of classification accuracy over Principle Components Analysis−Back Propagation neutral network (PCA-BPNN) and Moving Average−Linear Discriminant Analysis (MA-LDA). We found that the classification accuracy of the proposed classifier was greater than that of PCA-BPNN by 5%, and greater than that of LDA by 10.7%.

## 4. Conclusions

This paper presented a pattern recognition system for classifying ten brands of Chinese liquors based on MDS and SVM algorithms in the QCM-based e-nose we designed. We evaluated the comprehensive performance of the MDS-SVM classifier for predicting all ten brands of Chinese liquors individually. The prediction accuracy of the MDS-SVM classifier is superior to that of both the LDA and BP-ANN classifiers. Numerical experiment results of the classification of the ten brands of Chinese liquors showed that our recognition system is a viable solution for liquor classification problems. The proposed approach based on MDS and SVM applications has the following properties:

- Good accuracy at the reasonably small size of samples,
- Low calibration cost,
- Objective analysis and comprehensive assessment of Chinese liquors,
- Good adaptability and prediction (generalization) performance on general working conditions.

Conclusively, our proposed system may find practical application in Chinese liquors quality control and flavor assessment.

## Figures and Tables

**Table 1:** Details of the Chinese liquors used in our experiments.

No.	Liquors	Main Raw Materials	Fermentation Starter	Fermentation Duration	Aging Duration	Proof	Date	Flavor Type	Place of Origin
1	Fen Liquor	Sorghum	Daqu^†^	28 days	1 years	106	2011	Fen-flavor	Xinghua in Shanxi Province
2	GuoJiao1573	Sorghum, rice	Daqu	365 days	5 years	106	2011	Strong-flavor	Luzhou in Sichuan Province
3	Jiannanchun	Rice, Sorghum	Daqu	90 days	2 years	106	2011	Strong-flavor	Mianyang in Sichuan Province
4	Jiugui Liquor	Sorghum, rice, glutinous rice, maize	Xiaoqu^‡^	50 days	3 years	106	2011	Mixed-flavor	Jishou in Hunan Province
5	Kouzi Cellar	Sorghum, wheat, rice, pea	Daqu	35 days	2 years	106	2011	Mixed-flavor	Suixi in Anhui Province
6	Moutai	Sorghum, wheat, rice,	Daqu	210 days	3 years	106	2011	Moutai-flavor	Maotai in Guizhou Province
7	NiuLanshan	Sorghum, wheat	Daqu	30 days	1 year	106	2011	Strong-flavor	Beijing
8	Shuijingfang	Sorghum, wheat, maize, glutinous rice, rice,	Daqu	180 days	2 years	106	2011	Strong-flavor	Chengdu in Sichuan Province
9	Wuliangye	Sorghum, rice, glutinous rice, wheat, maize	Daqu	70 days	3 years	106	2011	Strong-flavor	Yibin city in Sichuan Province
10	XifengLiquor	Sorghum, wheat	Daqu	18 days	2 years	106	2011	Special-flavour	Baoji in Shanxi Province

^†^ Daqu is a type of grain, qu, which is made from raw wheat, barley, and/or peas [17]. ^‡^ Compared to daqu, xiaoquis a small starter, which is made from rice or rice bran [18].

**Table 2:** Composition of the eight sensor coatings in the sensor array.

Thin Coating	Composites	Thin Coating	Composites
Coating-1	PVC	Coating-5	AgCl
Coating-2	Polyamide	Coating-6	Azithromycin
Coating-3	Polyethylene (PE) + AgCl	Coating-7	CuCl_2_ + PE
Coating-4	Polytef	Coating-8	CuCl_2_ + AgCl + PE

**Table 3:** Group of raw data of the Moutai sample’s characteristic information.

No	1	2	3	4	5	6	7	…	200
Sensor-1	9,995,162	9,995,166	9,995,167	9,995,161	9,995,161	9,995,161	9,995,162	…	9,996,184
Sensor-2	9,996,246	9,996,245	9,996,246	9,996,245	9,996,254	9,996,245	9,996,245	…	9,996,255
Sensor-3	9,997,116	9,997,116	9,997,120	9,997,124	9,997,116	9,997,115	9,997,120	…	9,997,115
Sensor-4	9,995,703	9,995,697	9,995,700	9,995,697	9,995,697	9,995,697	9,995,697	…	9,995,698
Sensor-5	9,995,673	9,995,682	9,995,675	9,995,675	9,995,681	9,995,681	9,995,680	…	9,995,680
Sensor-6	9,995,896	9,995,896	9,995,896	9,995,896	9,995,906	9,995,896	9,995,896	…	9,995,896
Sensor-7	9,994,382	9,994,382	9,994,378	9,994,384	9,994,380	9,994,379	9,994,380	…	9,994,384
Sensor-8	9,993,190	9,993,191	9,993,191	9,993,197	9,993,193	9,993,191	9,993,197	…	9,993,193

**Table 4:** Classification results of Chinese liquor samples by leave-one-out cross-validationand 5-fold cross-validation.

No.	Liquor Name	Leave-One-Out Cross-Validation			5-Fold Cross-Validation		
		Num. of Errors	Num. of Samples	Accuracy	Num. of Errors	Num. of Samples	Accuracy
1	Fen Liquor	0	8	100%	0	8	100%
2	GuoJiao1573	1	8	87.5%	0	8	100%
3	Jiannanchun	1	8	87.5%	2	8	75%
4	Jiugui Liquor	0	8	100%	0	8	100%
5	Kouzi cellar	0	8	100%	0	8	100%
6	Moutai	0	8	100%	0	8	100%
7	Niulanshan	0	8	100%	0	8	100%
8	Shuijingfang	1	8	87.5%	1	8	87.5%
9	Wuliangye(liquor)	0	8	100%	0	8	100%
10	Xifeng Liquor	0	8	100%	0	8	100%
Total		3	80	96.25%	3	80	96.25%

**Table 5:** Classification results of the Chinese liquor samples by means of the e-nose and the MDS-SVM classifier.

Actual Brand (Liquor Name)	Mispredicted Brand (Liquor Name)	Num. of Errors	Num. of Samples	Accuracy
Fen Liquor	-	0	30	100%
GuoJiao1573	Shuijingfang	2	30	93.4%
Jiannanchun	Shuijingfang/GuoJiao1573	1/1	30	93.4%
Jiugui Liquor	-	0	30	100%
Kouzi cellar	-	0	30	100%
Moutai	-	0	30	100%
Niulanshan	-	0	30	100%
Shuijingfang	Jiannanchun	1	30	96.7%
Wuliangye	-	0	30	100%
Xifeng Liquor	-	0	30	100%
Total		5	300	98.3%

**Table 6:** Comparison of Classification Ability.

Method	Classification Accuracy
MDS-SVM	98.3%
PCA-BPNN	93.3%
MA-LDA	87.6%
